# The biomedical engineer’s pledge: overview and context

**DOI:** 10.1007/s11517-025-03443-6

**Published:** 2025-09-24

**Authors:** Antoni Ivorra, Txetxu Ausín, Laura Becerra-Fajardo, Antonio J. del Ama, Jesús Minguillón, Aracelys García-Moreno, Jordi Aguiló, Filipe Oliveira Barroso, Bart Bijnens, Oscar Camara, Sara Capdevila, Roger Castellanos Fernandez, Rafael V. Davalos, Jean-Louis Divoux, Ahmed Eladly, Dario Farina, Carla García Hombravella, Raquel González López, Cesar A. Gonzalez, Jordi Grífols, Felipe Maglietti, Shahid Malik, Elad Maor, Guillermo Marshall, Berta Mateu Yus, Lluis M. Mir, Juan C. Moreno, Xavier Navarro, Núria Noguera, Andrés Ozaita, Gemma Piella, José L. Pons, Rita Quesada, Pilar Rivera-Gil, Boris Rubinsky, Aurelio Ruiz Garcia, Albert Ruiz-Vargas, Maria Sánchez Sánchez, Andreas Schneider-Ickert, Ting Shu, Rosa Villa Sanz, Bing Zhang, Gema Revuelta

**Affiliations:** 1 Universitat Pompeu Fabra, Barcelona, Spain; 2 Serra Húnter Fellow Programme, Universitat Pompeu Fabra, Barcelona, Spain; 3 Instituto de Filosofía, Spanish National Research Council (CSIC), Madrid, Spain; 4 Rey Juan Carlos University, Madrid, Spain; 5 Universitat Autònoma de Barcelona, Bellaterra, Barcelona, Spain; 6 Centro de Investigación Biomédica en Red de Bioingenieria, Biomateriales y Nanomedicina (CIBER BBN), Saragossa, Spain; 7 Cajal Institute, Spanish National Research Council (CSIC), Madrid, Spain; 8 ICREA, Barcelona, Spain; 9 Comparative Medicine and Bioimage Centre (CMCiB), Germans Trias i Pujol Research Institute, Badalona, Barcelona, Spain; 10 Wallace H. Coulter Department of Biomedical Engineering, Georgia Tech—Emory University, Atlanta, GA USA; 11 Advice In Medical Device (A!MD), Besançon, France; 12 University of Manchester, Manchester, UK; 13 Department of Bioengineering, Imperial College London, London, UK; 14 Universitat de Barcelona, Barcelona, Spain; 15 Universiteit Utrecht, Utrecht, Netherlands; 16 Instituto Politécnico Nacional, Mexico, Mexico; 17 Instituto Universitario de Ciencias de la Salud, Fundación Barceló-CONICET, Buenos Aires, Argentina; 18 Indian Institute of Technology Delhi, Delhi, India; 19 Sheba Medical Center, Ramat Gan, Israel; 20 Tel Aviv University, Tel Aviv, Israel; 21 Facultad de Ciencias Exactas y Naturales, Instituto de Física Interdisciplinaria y Aplicada, Universidad of Buenos Aires, Buenos Aires, Argentina; 22 Consejo Nacional de Investigaciones Científicas y Técnicas, Buenos Aires, Argentina; 23 Universitat Politècnica de Catalunya, Barcelona, Spain; 24 CNRS, Gustave Roussy, Metabolic and Systemic Aspects of Oncogenesis (METSY), Université Paris-Saclay, Villejuif, Paris, France; 25 Center for Automation and Robotics (CAR), Spanish National Research Council (CSIC), Madrid, Spain; 26 Institute of Neuroscience and Dept Cell Biology, Physiology, and Immunology, Universitat Autònoma de Barcelona, Bellaterra, Barcelona, Spain; 27 Institute Guttmann of Neurorehabilitation, Badalona, Barcelona, Spain; 28 Tecnologia Regenerativa Qrem S.L., Barcelona, Spain; 29 Shirley Ryan AbilityLab, Chicago, IL USA; 30 Northwestern University, Evanston, IL USA; 31 University of California Berkeley, Berkeley, CA USA; 32 Fraunhofer Institute for Biomedical Engineering (IBMT), Sulzbach, Saarbrücken, Germany; 33 Instituto de Microelectrónica de Barcelona IMB-CNM, CSIC, Bellaterra, Barcelona, Spain; 34 Intelligent Energy-Based Tumor Ablation Laboratory, School of Mechatronic Engineering and Automation, Shanghai University, Shanghai, China; 35 Science, Communication, and Society Studies Center, Universitat Pompeu Fabra, Barcelona, Spain

**Keywords:** Biomedical engineering, Ethics, Pledge, Hippocratic Oath

## Abstract

Although biomedical engineering (BME) is a profession with ethical responsibilities comparable to those in medicine, it has, until now, lacked a counterpart to the Hippocratic Oath. While professional societies have established codes of ethics for biomedical engineers, these documents lack the symbolic and ceremonial significance of an oath or pledge. By contrast, the recitation of the Hippocratic Oath, or its modern version, the “Physician’s Pledge,” serves as a powerful rite of passage for medical students, fostering a strong sense of ethical duty at the start of their professional journey. However, the content of the Hippocratic Oath includes elements specific to clinical practice and is not directly applicable to biomedical engineering. To fill this gap, we have created a “Biomedical Engineer’s Pledge,” comprising a preamble, ten promises, and a concluding statement, to inspire ethical awareness and establish a meaningful graduation tradition.

**Graphical abstract:**

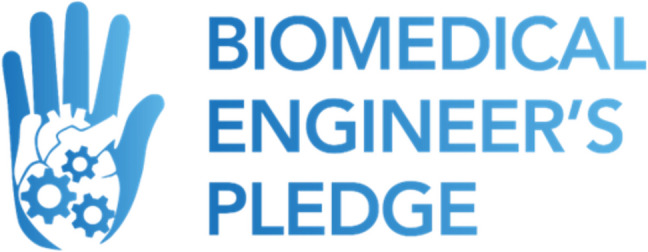

## Introduction

Biomedical engineers operate at the intersection of healthcare and technology, where ethical dilemmas frequently arise. In medicine, a symbolic yet essential element in shaping ethical awareness is the Hippocratic Oath, whose origins can be traced back to ancient Greece [1]. Its modern adaptation, the World Medical Association’s “Physician's Pledge” or Declaration of Geneva, continues this tradition as a brief, non-binding declaration taken at graduation [2]. Pledges and oaths offer a compelling way to cultivate ethical awareness through a memorable symbolic act that requires little academic effort. Regrettably, the content of the “Physician's Pledge” includes elements specific to clinical practice and is not directly applicable to biomedical engineering. To fill this gap, we created the “Biomedical Engineer’s Pledge,” modeled after the “Physician’s Pledge” but adapted to the unique ethical landscape of the discipline, while explicitly introducing some additional aspects (e.g., the avoidance of fraud against patients and the minimization of animal experimentation). This effort involved students, academics, industry professionals, physicians, and ethicists.

In the past, calls have been made for the implementation of a “Hippocratic Oath” for biomedical engineering [3], and several initiatives in this direction have been undertaken. These include dissertations proposing ethical guidelines for biomedical engineers [4], ethical codes by biomedical engineering societies [5–9], and solemn declarations such as the Order of the Engineer taken by graduating students “to serve as ethical engineers” [10] or “pledges to professionalism” [11], even including the performance of very ceremonial acts involving tokens such as rings [12]. Remarkably, France has recently introduced a mandatory research integrity oath as a requirement for obtaining a PhD [13]. However, to the best of our knowledge, nothing resembling an oath or pledge specifically aimed at biomedical engineering students has ever been published.

The process used to collaboratively draft the pledge, along with a detailed explanation of its wording and principles, is documented in a manuscript available in the public repository Zenodo [14]. More interestingly, the manuscript also includes several case studies or ethical dilemmas, outlined to illustrate how the pledge can be applied in practice.

We believe the pledge will not only reinforce ethical values among students but also strengthen public understanding of biomedical engineering, foster interprofessional solidarity, and serve as a source of pride for students and their families.

## The pledge

I solemnly declare that as a biomedical engineer:

1. I will hold paramount the safety, health, and well-being of patients, research participants, coworkers, healthcare workers, and the public.
2. Upholding the above, I will exercise my profession with integrity and responsibility.
3. I will do my best to ensure the autonomy and dignity of patients and research participants.
4. I will ensure the proper safeguarding of patient and research participant data.
5. I will not discriminate on the grounds of age, sex, sexual orientation, gender identity or expression, disease, functional diversity, origin, racial status, religious beliefs, political affiliation, social class, or any other identity factor.
6. I will not participate in patient deception or fraud against them.
7. I will share my scientific and technical knowledge, and I will not use it to violate human rights.
8. I will promote the replacement, reduction, and refinement of the use of animals in research.
9. I will contribute to the environmental and economic sustainability of healthcare and to universal health coverage.
10. I will demonstrate to my teachers, colleagues of any discipline, students, and society at large, the respect and gratitude that is their due.

I understand and commit myself freely and publicly to these principles.

## Ethical foundations of the pledge

The pledge is structured around ten commitments, or promises, with the first commitment prioritizing the safety, health, and well-being of patients, research participants, coworkers, healthcare workers, and the public. As in the case of the “Physician’s Pledge”, the “Biomedical Engineer’s Pledge” can be fitted to the general bioethics framework of the Four Principles devised by Beauchamp and Childress: beneficence, justice, non-maleficence, and respect for autonomy [15]. However, the four general principles are not considered equally important in the pledge: non-maleficence and beneficence are given top priority in the first commitment. This same combination of the fundamental principles of beneficence and non-maleficence is captured in the classical formulation by Thomas Aquinas, “bonum est faciendum et malum vitandum,” meaning “good is to be done and evil is to be avoided” [16].

The specification of beneficiaries (i.e., “patients, research participants, coworkers, healthcare workers, and the public”) serves to prevent oversights, and equally important is the clear identification of the purposes (“safety, health, and well-being”). Although no explicit hierarchy is stated, it is by no coincidence that safety comes first. This aligns with the Latin phrase “Primum non nocere” (“Above all, do no harm”), which underscores the principle of non-maleficence. Widely taught in medical schools, this aphorism has uncertain origins and is often mistakenly attributed to the Hippocratic Oath [17].

It is important to note that the explicit prioritization of the first promise implicitly demands an assessment of the potential consequences of actions or omissions. In other words, a careful analysis of risks and benefits is required. Actions must be evaluated by weighing their risks against their anticipated benefits. This benefit-risk consideration is a cornerstone of guidelines followed by ethics committees overseeing medical and human research. Notably, the “Nuremberg Code” for medical research, developed in response to Nazi atrocities, states: “The degree of risk to be taken should never exceed that determined by the humanitarian importance of the problem to be solved by the experiment” [18].

The second commitment or promise—“Upholding the above, I will exercise my profession with integrity and responsibility”—emphasizes the moral obligations of biomedical engineers, though it holds secondary priority to the first promise. Given the potential life-altering impacts of biomedical engineering, practitioners must accept accountability for their actions and outcomes. Ethical responsibility extends beyond legal compliance, requiring awareness of consequences and a commitment to “doing the right thing.” Integrity involves consistently upholding high moral and professional standards, supported by good practices. The subsequent promises should be understood as specific expressions of what it means to act with integrity and responsibility. As a guiding principle, provided the first promise remains uncompromised, these later commitments should take precedence over other moral considerations.

Biomedical engineering is a rapidly advancing field where technological developments must be balanced with ethical considerations. The pledge explicitly acknowledges the risk of fraud, deception, and unethical manipulation of medical technology, reinforcing a standard of accountability. The Theranos case, in which misleading claims led to severe ethical violations, serves as a cautionary tale, illustrating the real-world implications of ethical lapses in biomedical engineering [19].

The “Biomedical Engineer’s Pledge” draws inspiration from the “Physician’s Pledge” and from ethical codes developed by biomedical engineering societies such as the Code of Ethics of the Biomedical Engineering Society (BMES) [6], the Canadian Medical and Biological Engineering Society Code of Ethics [8], and the IEEE Engineering in Medicine and Biology Society (EMBS) Code of Ethics [9]. As a result, it shares important formal and conceptual similarities, particularly in relation to the fundamental ethical principles discussed above. However, the pledge also explicitly introduces certain ethical aspects that are overlooked or not fully addressed in some of these existing sources. For instance, the pledge also addresses issues such as patient autonomy, safeguarding of medical data, and non-discrimination. These aspects align with broader societal concerns, ensuring that biomedical engineering students understand the importance of ethical considerations in research and design. In a data-driven world, where neurotechnologies, medical records, and AI-based diagnostics are becoming prevalent, it is crucial for students to adopt strict ethical guidelines regarding data security and patient confidentiality from the very beginning of their careers.

Another notable and relatively overlooked aspect of the pledge is its emphasis on environmental and economic sustainability. Biomedical engineering, while transformative, also contributes to medical waste, carbon emissions, and economic disparities in healthcare accessibility. The pledge urges students to consider the long-term impact of medical innovations, ensuring that healthcare systems remain sustainable and inclusive.

Furthermore, as an additional novel element, the pledge promotes universal healthcare coverage, urging students to develop technologies that can bridge healthcare disparities rather than exacerbate them. This principle aligns with global health initiatives that seek to expand access to medical devices and treatments in underserved communities. The pledge, therefore, extends beyond individual responsibility to a collective ethical duty to advance global healthcare.

## Use of the pledge and endorsements

Ethical training is often underemphasized in biomedical engineering curricula, with many programs offering only limited exposure to bioethics. The “Biomedical Engineer’s Pledge” provides an opportunity to institutionalize ethical awareness, similar to how the “Physician’s Pledge” has shaped medical professionals’ ethical standards. By incorporating the pledge into graduation ceremonies or professional milestones, institutions can foster a culture of ethical consciousness among biomedical engineering students. Although the pledge is primarily intended to be taken as a rite of passage by BSc students around their graduation, its preamble sentence is intentionally short and generic to allow the use of the pledge across various educational levels (BSc, MSc, and PhD) and contexts (e.g., before graduation, at the graduation ceremony, just after graduation, or many years after graduation, for instance, at alumni reunions).

Furthermore, adopting the pledge at an early stage in education helps students internalize ethical principles before they enter the workforce. It serves as a guiding framework for their decision-making, ideally through their entire careers. The public and voluntary nature of the pledge further enhances accountability, encouraging students to uphold their commitments beyond academic settings.

Many students who earn degrees in biomedical engineering go on to pursue careers in fields unrelated to biomedical engineering. However, even in such cases, the pledge can still serve as a valuable moral compass, guiding their professional conduct and ethical decision-making regardless of the specific discipline they ultimately follow.

The pledge has already been recited three times at the graduation ceremonies of biomedical engineering students at Universitat Pompeu Fabra in Barcelona, Spain, and once at the graduation ceremony at Universidad Politécnica de Cartagena in Cartagena, Spain.

Notably, the “Biomedical Engineer’s Pledge” has been officially endorsed by the Hippocratic Movement, a non-profit organization dedicated to promoting Hippocratic ideals among healthcare professionals, and it was also endorsed for recitation in July 2024 by the International Federation for Medical & Biological Engineering.

## Conclusion

The “Biomedical Engineer’s Pledge” represents a significant step toward formalizing ethical responsibility within the biomedical engineering profession, with a particular emphasis on students. By outlining clear ethical commitments—ranging from patient safety to sustainability and global health equity—the pledge serves as a moral compass for both aspiring and experienced biomedical engineers. Unlike traditional regulatory frameworks, this pledge operates on a voluntary commitment, reinforcing the idea that ethical practice should be intrinsic rather than imposed. If widely adopted in educational institutions, the “Biomedical Engineer’s Pledge” has the potential to elevate the profession’s ethical standards, enhance public trust, and ensure that biomedical innovations continue to serve humanity responsibly.

The “Biomedical Engineer’s Pledge” can be accessed at https://www.upf.edu/web/biomedical-engineers-pledge and is licensed under a Creative Commons Attribution-NoDerivatives 4.0 International License.

## Appendix

Spanish, French, and Catalan versions of the pledge

“Compromiso de la ingeniera biomédica y del ingeniero biomédico”

Declaro solemnemente que como profesional de la ingeniería biomédica:

1. Tendré como prioridad la seguridad, la salud y el bienestar de pacientes, participantes en estudios de investigación, colegas de trabajo, personal sanitario y el público.
2. Respetando lo anterior, ejerceré mi profesión con integridad y responsabilidad.
3. Haré todo lo posible para garantizar la autonomía y la dignidad de pacientes y participantes en estudios de investigación.
4. Garantizaré la adecuada salvaguarda de los datos de pacientes y participantes en estudios de investigación.
5. No discriminaré por motivos de edad, sexo, orientación sexual, identidad o expresión de género, enfermedad, diversidad funcional, origen, condición racial, creencias religiosas, filiación política, clase social o cualquier otro factor de identidad.
6. No participaré en engaño o fraude a pacientes.
7. Compartiré mis conocimientos científicos y técnicos, y no los utilizaré para violar los derechos humanos.
8. Promoveré el reemplazo, la reducción y el refinamiento en el uso de animales en la investigación.
9. Contribuiré a la sostenibilidad medioambiental y económica de la asistencia sanitaria y a la cobertura sanitaria universal.
10. Demostraré a mi profesorado, colegas de cualquier disciplina, estudiantes y a la sociedad en general, el respeto y la gratitud que les corresponde.

Entiendo y me comprometo libre y públicamente con estos principios.

“Engagement de l’ingénieure biomédicale et de l’ingénieur biomédical”

Je déclare solennellement qu’en tant que professionnelle ou professionnel de l’ingénierie biomédicale:

1. Ma priorité sera la sécurité, la santé et le bien-être des patients, des participants à des recherches biomédicales, des collègues de travail, des personnels de santé et du public.
2. Dans ce cadre, j’exercerai ma profession avec intégrité et responsabilité.
3. Je ferai tout ce qui est en mon pouvoir pour garantir l’autonomie et la dignité des patients et des participants à des recherches biomédicales.
4. J’assurerai la protection des données concernant les patients et les participants à des recherches biomédicales.
5. Je ne ferai aucune discrimination fondée sur l’âge, le sexe, l’orientation sexuelle, l’identité ou l’expression de genre, la maladie, la diversité fonctionnelle, l’origine, la race, les croyances religieuses, l’affiliation politique, la classe sociale ou tout autre facteur identitaire.
6. Je ne participerai à aucune tromperie ou fraude envers les patients.
7. Je partagerai mes connaissances scientifiques et techniques et je ne les utiliserai pas pour violer les droits humains.
8. J’encouragerai le remplacement, la réduction et le raffinement des expériences conduites avec des animaux.
9. Je contribuerai à la durabilité environnementale et économique des soins de santé et à la couverture sanitaire universelle.
10. Je montrerai à mes professeurs, à mes collègues de toute discipline, aux étudiants et à la société en général, le respect et la gratitude qui leur revient.

Je comprends et je m’engage librement et publiquement à respecter ces principes.

“Compromís de l’enginyera biomèdica i de l’enginyer biomèdic”

Declaro solemnement que com a professional de l’enginyeria biomèdica:

1. Tindré com a prioritat la seguretat, la salut i el benestar de pacients, participants en estudis de recerca, col·legues de feina, treballadors sanitaris i el públic.
2. Respectant això, exerciré la meva professió amb integritat i responsabilitat.
3. Faré tot el possible per garantir l’autonomia i la dignitat de pacients i participants en estudis de recerca.
4. Garantiré la salvaguarda adequada de les dades de pacients i participants en estudis de recerca.
5. No discriminaré per motius d’edat, sexe, orientació sexual, identitat o expressió de gènere, malaltia, diversitat funcional, origen, condició racial, creences religioses, filiació política, classe social o qualsevol altre factor d’identitat.
6. No participaré en engany o frau a pacients.
7. Compartiré els meus coneixements científics i tècnics, i no els faré servir per violar els drets humans.
8. Promouré el reemplaçament, la reducció i el refinament en l’ús d’animals en la recerca.
9. Contribuiré a la sostenibilitat ambiental i econòmica de l’assistència sanitària i a la cobertura sanitària universal.
10. Demostraré al meu professorat, col·legues de qualsevol disciplina, estudiants i a la societat en general, el respecte i la gratitud que els correspon.

Entenc aquests principis i m’hi comprometo lliurement i públicament.
